# The impact of armed conflict in eastern Democratic Republic of Congo on the risk of malaria outbreaks in children under five years old

**DOI:** 10.1016/j.nmni.2025.101636

**Published:** 2025-09-02

**Authors:** Hermann Yokolo, Christian Tague, Joshua Ekouo, Dujardin Makeda, Aymar Akilimali

**Affiliations:** Department of Research, Medical Research Circle (MedReC), Goma, Democratic Republic of the Congo

**Keywords:** Malaria, Epidemiology, Infection, Prevention, Democratic Republic of Congo

Dear editor

For several years, the Democratic Republic of Congo (DRC) has been plagued by recurring armed conflicts, leading to large-scale population displacements. This social and economic instability has deteriorated living conditions, especially in displacement camps, and has had severe public health consequences, including a resurgence of malaria. The displacement of vulnerable populations, combined with limited access to healthcare, facilitates the spread of malaria, a disease endemic to the region. In displacement camps, overcrowding, poor management of stagnant water, and the lack of sanitation infrastructure create favorable conditions for the proliferation of vector mosquitoes. This letter aims to highlight the indirect effects of armed conflicts on public health, focusing on the malaria outbreak among children under five, who are particularly affected by this crisis [1].

In the DRC, malaria affects approximately 95 million people. According to data from the Ministry of Public Health, Hygiene and Social Welfare's epidemiological surveillance system and the 2022 annual report of the National Malaria Control Program, children under five account for 50 % of malaria cases and nearly 70 % of malaria-related deaths. In 2022 alone, the country recorded 27,296,419 malaria cases, including 13,300,804 cases in children under five (48.7 %), with 1,176,648 cases classified as severe. Overall, more than 24,880 malaria-related deaths were recorded that year, including 16,921 among children under five (68 %) [1,2].

Children under five are a particularly vulnerable group and remain the most heavily impacted. Severe cases often require hospitalization and can involve complications such as severe anemia and convulsions. Conditions in displacement camps significantly favor disease transmission: the absence of treated mosquito nets, overcrowding, and poor wastewater management create ideal breeding grounds for *Anopheles* mosquitoes. Conflict-related barriers to treatment and healthcare access further exacerbate the situation. The destruction or abandonment of health facilities, the departure of healthcare personnel, and the disruption of essential services hinder the implementation of malaria control measures such as mosquito net distribution, public awareness campaigns, diagnosis, treatment, and indoor residual spraying. Without early treatment, mild cases frequently progress to severe forms, increasing mortality rates among children. Insecurity restricts the movement of humanitarian organizations and public health authorities, complicating the distribution of antimalarial drugs, diagnostic tests, mosquito nets, and other vital resources, thereby intensifying shortages in prevention and treatment [3,4].

Displaced populations often seek refuge in precarious shelters, frequently located near stagnant water sources—ideal mosquito breeding grounds. These poorly protected living spaces expose children under five to higher risk of mosquito bites, making them primary victims of malaria. Moreover, migration flows to other regions of the DRC contribute to the wider spread of malaria parasites. Infected individuals may introduce the disease into previously low-transmission areas, placing children with limited immunity at greater risk. Thus, ongoing conflict in eastern DRC significantly exacerbates malaria outbreaks, particularly among young children. The destruction of infrastructure, restricted access to healthcare, overcrowding, and the proliferation of mosquito breeding sites all contribute to increased transmission. This context underscores the indirect yet devastating effects of security crises on public health [1,3,5].

In response to this emergency, it is imperative to strengthen humanitarian aid efforts, restore healthcare services, and implement sustainable prevention strategies. Protecting the most at-risk children and containing the spread of malaria is not only a public health priority but a moral imperative in the face of an ongoing humanitarian crisis.

## Data availability

Not applicable.

## Ethics approval and consent to participate

Not applicable.

## Use of artificial intelligence tools

No artificial intelligence was used in generating the manuscript.

## Provenance and peer review

Not commissioned, externally peer reviewed.

## Supplementary information

Not applicable.

## CRediT authorship contribution statement

**Hermann Yokolo:** Supervision, Visualization, Writing – review & editing, Conceptualization, Validation, Writing – original draft. **Christian Tague:** Writing – review & editing, Conceptualization. **Joshua Ekouo:** Writing – review & editing. **Dujardin Makeda:** Writing – review & editing. **Aymar Akilimali:** Supervision, Visualization, Conceptualization, Validation.

## Funding

The authors did not receive any financial support for this work. No funding has been received for the conduct of this study.

## Declaration of competing interest

The authors declare that there no conflict of interest.
